# PD-1/PD-L1 pathway in non-small-cell lung cancer and its relation with EGFR mutation

**DOI:** 10.1186/s12967-014-0373-0

**Published:** 2015-01-16

**Authors:** Mei Ji, Yan Liu, Qing Li, Xiao-Dong Li, Wei-Qing Zhao, Hanze Zhang, Xiaofei Zhang, Jing-Ting Jiang, Chang-Ping Wu

**Affiliations:** Department of Oncology, The Third Affiliated Hospital of Soochow University, Changzhou, China; Department of Hematology, The Third Affiliated Hospital of Soochow University, Changzhou, China; Department of Pathology, The Third Affiliated Hospital of Soochow University, Changzhou, China; Department of Epidemiology and Biostatistics, College of Public Health, University of South Florida, Tampa, USA; Department of Clinical Oncology, Institute of Development, Aging and Cancer, Tohoku University, Sendai, Japan; Department of Biological Treatment, The Third Affiliated Hospital of Soochow University, Changzhou, China

**Keywords:** PD-1, PD-L1, Non-small-cell lung cancer, EGFR, Gene mutation

## Abstract

Immunotherapy has become a crucial modality for non-small-cell lung cancer treatment. Recently, two immune checkpoints, PD-1 and PD-L1, have emerged as important targets for immunotherapy. Their antitumor efficacy has been confirmed by in vitro and in vivo studies. But the correlation between PD-1/PD-L1 expression and EGFR expression was controversial and needs more evidences to support the combination of PD-1/PD-L1 inhibitors and tyrosine kinase inhibitors.

## Introduction

Lung cancer, the leading cause of cancer-related deaths, has the highest incidence and mortality rates amongst all malignancies worldwide [1]. Lung cancer has an incidence of over 1.6 million cases/year accounting for 13% of all new cancer diagnoses and 1.4 million deaths/year accounting for 18% of all cancer-related deaths [2,3]. Particularly in China, both of the incidence and mortality rates of lung cancer are the highest over the world [4].

Amongst the various types of lung caners, non-small-cell lung cancer (NSCLC) comprises 80% - 85% of all cases and more than 70% are diagnosed as unresectable advanced disease [1,5]. Though much progress has been obtained in optimizing the treatment of NSCLC, the prognosis for NSCLC patients remains poor, with the 5-year overall survival (OS) rate of 15% of all stages [1].

Up to date, the pivot for curing advanced NSCLC has been the direct inhibition of tumor cell growth by cytotoxic agents or targeted small-molecule inhibitors. However, platinum-based chemotherapy only has a response rate of 20% - 35% and a median OS of 10–12 months, while targeted therapy with tyrosine kinase inhibitors (TKIs) can not prolong OS significantly [6] and moreover eventual relapse or progression is inevitable. Hence, novel treatment strategies are urgently needed.

Given that cancers are recognized by human immune system, and under certain circumstances the immune system can obliterate tumors [7], many clinical physicians have considered immunotherapy as a treatment modality for NSCLC over the last several decades. It has already been found that chronic inflammation increases neutrophils, decreases lymphocytes, and increases cytokine release and interleukin secretion to reduce cancer cell apoptosis in lung [8]. Until recently, one promising breakthrough that NSCLC responds to immune checkpoint blockade has emerged, suggesting that NSCLC is an immunologically targetable disease.

T cells play pivotal effector-like roles in the complicated network of human the immune system. T cells hamper tumor development [9,10] but unfortunately tumors can also prevent themselves from sustained T cell responses via so called immune checkpoints like CTLA-4, PD-1 and PD-L1 etc. [11]. Studies in mouse models have revealed that manipulation of inhibitory immune checkpoints could reduce T cell responses against tumors [12]. In additon, we know that NSCLC induces pro-tumorigenic immunosuppressive changes to evade the immune system, and these changes can be elicited by the inhibitors [13]. So immune checkpoint inhibitors can restore T cell resoponses and thus impede the tumor evasion of NSCLC.

### Mechanisms of PD-1 and PD-L1 as cancer immunotherapy

PD-1, a type 1 transmembrane protein of the Ig superfamily [14], consists of an extracellular N-terminal IgV-like domain, a transmembrane domain, and a cytoplasmic tail [15] engaging in inhibitory signal transmission [16]. Being expressed on activated immune cell types including T cells, B cells, natural killer (NK) cells, NKT cells, dendritic cells (DCs), macrophages, and host tissues [17,18], the expression on effector T cells is associated with constitutive antigen exposure and thus PD-1 has become a marker of T cell unresponsiveness or exhaustion [19].

PD-1 has two known ligands, PD-L1 (B7-H1) and PD-L2 (B7-DC), which belong to B7 family [20,21]. PD-L1 is the major ligand and expressed on hematopoietic cells including T cells, B cells, DCs, macrophages and mast cells [22] as well as many nonhematopoietic cells including endothelial cells and numerous epithelial cells [23]. PD-L1 is expressed on many tumors including cancers developing in various organs such as head and neck, lung, stomach, colon, pancreas, breast, kidney, bladder, ovary, cervix, as well as melanoma, glioblastoma, multiple myeloma, lymphoma, and various leukemias [24-28], thereby inhibits effective anti-tumor immune responses mediated by PD-1-expressed T cells [11].

PD-1 and PD-1 L interact with each other and then inhibit expression of multiple transcription factors of T cells such as GATA-3 and T-bet [29]. Besides, the PD-1/PD-L1 interaction inhibits the proliferation, survival, and effector function of CD8^+^ cytotoxic T lymphocyte (CTL) and thus induces apoptosis of tumor-infiltrating T cells [30]. Ectopic PD-L1 expression in tumor cells in a syngeneic transplant model facilitated the escape of the tumor cells from CTL control [31].

Besides, it was shown that PD-L1 expression plays a critical role in differentiation of regulatory T cells (Tregs) and maintaining their suppressive function [32]. Because Tregs are important inhibitors of tumor-specific immune responses in the tumor microenvironment, PD-1/PD-L1-mediated generation of Tregs can help as another layer of protection to immune evasion of tumors. Therefore, blockage of PD-1 or PD-L1 can activate the anti-tumor activity through both effector T cell activation and Treg inhibition. Additionally, PD-1/PD-L1 interactions can promote the differentiation of CD4^+^ T cells into FOXP3^+^ Tregs [30], further suppressing the immune system and resulting in peripheral immune tolerance in cancer patients [33]. As well, the effects of PD-1 blockade can be mediated partially by B cells or NK cells. An in vitro study revealed that PD-1 could inhibit stimulating signals of B cell receptors, leading to restoration of B cell activation after transfecting gene fragments of PD-1 into B lymphoma cell lines [34]. It has been reported that B cell antigen receptor signal inhibits B cell proliferation and function by inducing PD-1 expression [34] and Tumor-produced IL-18 inhibits the function of NK cells via enhancing PD-1 expression [35].

In gene level, tumor cells can activate PD-L1 expression of via multiple oncogenic signaling pathways involving IFN-γ/JAK2/IFN [24], PI3K [36], ALK/STAT3 [37], MEK/ERK/STAT1 [38], MYD88/TRAF6 [38] or exposure to inflammatory cytokines such as IFN-γ [39] produced by infiltrating immune cells.

Altogether, PD-L1/PD-1 interation plays an important role in the reduction of specific T cell apoptosis, inhibition of immune response to tumors, and immune evasion of tumors [40]. The inhibition of PD-1/PD-L1 pathway may hamper the proliferation of activated effector T cell, causing the tumor evasion from the killing of CTLs, resulting in the weakening of anti-tumor immune response [41].

### PD-L1 blockade for cancer immunotherapy

Given that PD-1 is highly expressed on lymphocytes infiltrating human tumors and circulating tumor-specific T cells [42], and together with that PD-L1 expression is correlated with prognosis in many cancers, which suggests that PD-L1 expression is a mechanism for tumor immune evasion [24,28,31], it is reasonable to consider the blockade of the PD-1/PD-L1 interaction may be a promising anticancer immunotherapy.

PD-L1 overexpression on mouse mastocytoma models inhibited tumor-directed T cell cytotoxicity in vitro and furthermore promoted apoptosis of tumor-specific T cells and immune evasion, these effects were neutralized by antibody-mediated blockade of PD-L1 [24,31]. Moreover in several experiments, blockade of PD-L1 could promote immune-mediated destruction of tumors which expressed PD-L1 [24,43].

The clinical significance for prognosis of PD-L1 expression on tumors is controversial, correlating with inferior outcomes in some studies [44-47] and superior outcomes in others [39]. But the consensus is that there were no remarkable adverse immune-related events (irAE) reported in preclinical models of PD-1 or PD-L1 blockade. Overall, therapeutic blockade of PD-1 or PD-L1 might break these multiple layers of immune inhibition, allowing effective anti-tumor T cell responses.

### Studies in NSCLC

PD-1 overexpression on CD8^+^ T cells in NSCLC suggests a reduced production of various cytokines and T cell proliferation [48]. Abnormal expression PD-L1 has been identified in a range of 19% to 100% of NSCLC tumor cases [49-52], and associated with poor prognosis [50,51]. On the other hand, PD-L1^+^ cells are remarkably increased comparing with adjacent lung parenchyma, and PD-L1 expression on NSCLC cells also correlates with poor prognosis and shortened OS [48]. Hence it can be indicated that blockade of PD-1 interactions allows the tumor-specific T cell to unleash its full armamentarium of effector function on the NSCLC cells.

To date, a few antibodies which inhibit the PD-1 pathway by blockade of the PD-1 or PD-L1 have been invented.

#### Nivolumab

Nivolumab (BMS-936558) is a fully human IgG4 antibody blocking the PD-1 receptor [53]. Results of a phase I trial revealed an objective response rate (ORR) of 17% in 129 previously treated patients with advanced NSCLC [54]. Although the median PFS in the cohort was 2.3 months and the median OS was 9.9 months, these who responded enjoyed sustained benefits. Specifically, the 2-year OS rate was 24%, and many remained in remission after completing 96 weeks of continuous therapy.

The most critical clinical data of nivolumab is from a phase Ib dose-escalation study enrolling 269 patients with various advanced solid tumors including NSCLC [55]. A promising response rate of 18% was observed in NSCLC patients. Rates of irAE (diarrhea, rash) appeared to be few and tolerable.

In other studies, nivolumab has also been tested in combination with platinum for first-line NSCLC, with an ORR of 33%, and a grade 3 or 4 AE rate of 49%, and most of AEs were attributable to chemotherapy [56].

In the above trials, patients generally received anti-PD-1 antibody until progression for 1 to 2 years. If study participants achieved a durable response and then subsequently progressed after cessation of therapy, there was an opportunity for rechallenge at the time of progression [57].

#### Lambrolizumab

Lambrolizumab (MK-3475) is a humanized IgG4 antibody directed against PD-1 [58]. In a phase I open-label, dose-escalation study enrolling advanced solid tumor patients, clinical activity of an unconfirmed partial response was observed in 1 patient with squamous NSCLC.

BMS-936559 (previously MDX-1105) is a fully human, PD-L1-specific IgG4 mAb [53]. A phase I study evaluating its anti-tumor activity reported an ORR of 10% in patients with NSCLC [59]. No cases of pneumonitis were reported.

### Correlation between PD-1/PD-L1 expression and EGFR/KRAS expression

In the clinical trials mentioned above, only a subset of patients responded to PD-1 blockade. And NSCLC is a disease characterized by driver mutation-defined molecular subsets, each with distinct clinicopathologic features and potentials for targeted therapies. Hence, it is appealing to explore the clinicopathological characteristics and molecular associations of NSCLC expressing PD-1 or PD-L1, which might be candidates for anti-PD-1/PD-L1 immunotherapy.

As widely known, one of the most commonly mutated oncogenes in NSCLC patients is EGFR, a member of receptor tyrosine kinases superfamily. EGFR binds to extracellular ligands, dimerizes with them and auto-phosphorylate [60]. Then, EGFR acts as a key regulatory molecule in cellular signaling pathways, promoting tumor cell proliferation, invasion and metastasis [61]. EGFR is over-expressed by 40 to 80% of NSCLC, and the expression levels are correlated with the EGFR tyrosine kinase domain mutations [62].

EGFR tyrosine kinase inhibitors (TKIs) have been developed for the treatment of advanced NSCLC for years [63]. Unfortunately, only one part of NSCLC patients is sensible to EGFR-TKIs. Despite the initial response, tumors become TKI-resistant by acquiring either a secondary point mutation in EGFR (T790M) or additional alterations in other genes that bypass the requirement for ongoing signaling from the mutated EGFR [64].

Preliminary results have suggested that PD-L1 expression might be associated with a higher likelihood of response to PD-1 blockade [65], but reliable biomarkers associated with treatment response remain poorly known. Activation of EGFR pathway might be involved in suppressing the immune response in murine melanoma models either through activating regulatory T cells or reducing the levels of the T cell chemoattractant [65]. Besides, other genes such as KRAS, BRAF and ALK are also important.

The results of the correlation between PD-1/PD-L1 expression and EGFR expression were controversial. Gettinger et al. concluded that EGFR or KRAS mutation status did not correlate with response rate of nivolumab for NSCLC patients [66]. Akbay et al.[67] found that activation of the EGFR pathway induced PD-L1 expression to help NSCLC tumors evade from the antitumor immune response. And it has been observed that EGFR signaling was independent of its effects on cell proliferation and survival. D’Incecco et al. [68] observed that PD-1 positive status was significantly associated with the presence of KRAS mutations (*P* = 0.006), while PD-L1 positive status was significantly associated with presence of EGFR mutations (*P* = 0.001). Patients with PD-L1 positive expression had higher sensitivity to EGFR-TKIs, longer time to progression and OS (*P* = 0.09) than PD-1 negative patients. Mu et al. observed that in stage I NSCLC patients the rate of PD-L1 over-expression was 39.9% (65/163) but there was no significant correlation between PD-L1 expression and EGFR/KRAS/BRAF/ALK expression [51]. Similarly, Zhang et al. found that there was no significant relations between PD-L1 expression and EGFR/KRAS expression in lung adenocarcinoma [69].

Conclusively, the mentioned genes, not only EGFR, but also other several genes such as KRAS, BRAF or ALK, can not be satisfactory biomarkers for assessing the effects of blockage of PD-1/PD-L1 pathway based on the results of existing studies.

### Future prospective

Nowadays, the urgent need-to-be solved problems include the start time point of antibodies using, the duration of administration, the combination with other treatment modalities, the identifying of priority population and the overcoming of antibody resistance [28].

Referring to the combination with other treatment modalities, the distinct molecular and cellular mechanisms whereby CTLA-4 and PD-1 suggest that combined therapeutic strategy of these pathways might be synergistic for cancer immunotherapy. Preclinical models of combined CTLA-4 and PD-1 blockade have shown promising results without a significant increase in toxicity [70]. The clinical activity of checkpoint-blocking antibodies in the monotherapy setting is established, and the following step is to assess the safety and activity of combinations.

One noticeable point is that the therapeutic mechanism of PD-1/PD-L1 targeted therapy is different from chemotherapy and present molecular targeted therapies. Hence, novel assessment criteria for therapeutic effects are needed. We have proposed a preliminary evalation system for the effect of another widely used antitumor immunotherapy, cytokine-induced killer cell therapy [71], and the system for PD-1/PD-L1 targeted therapy is presumed to be similar to some extent.

Furthermore, the relative contributions of T cells, B cells, NK cells, NKT cells, DCs, and macrophages to the anti-cancer activity of PD-1 blockade should be clarified more deeply, as PD-1 is expressed on all of these types of immune cells.

Conclusively, PD-1 and PD-L1 are important checkpoints in tumor development. PD-1/PD-L1 pathway is a promising target for treating NSCLC, and its correlation with EGFR/KRAS mutation needs confirmation by progressive studies.
